# Developing a New Expected Goals Metric to Quantify Performance in a Virtual Reality Soccer Goalkeeping App Called CleanSheet

**DOI:** 10.3390/s24237527

**Published:** 2024-11-25

**Authors:** Matthew Simpson, Cathy Craig

**Affiliations:** 1 School of Maths & Physics, Queens University Belfast, Belfast BT7 1NN, UK; msimpson22@qub.ac.uk; 2 School of Psychology, Ulster University, Cromore Road, Coleraine BT52 1SA, UK

**Keywords:** VR training, sports performance, xG models, goalkeeping

## Abstract

As virtual reality (VR) sports training apps start to become more mainstream, it is important that human performance is measured from VR gameplay interaction data in a more meaningful way. *CleanSheet* is a VR training app that is played by over 100,000 users around the world. Many of those players are aspiring goalkeepers who want to use the app as a new way to train and improve their general goalkeeping performance. Whilst the leaderboards display how many shots players saved, these data do not take into account the difficulty of the shot faced. This study presents a regression model developed from a combination of existing expected goals (xG) models, goalkeeper performance metrics, and psychological research to produce a new shot difficulty metric called CSxG. Utilizing user save rate data as the target variable, a model was developed that incorporated three input variables relating to ball flight and in-goal positioning. Our analysis showed that the required rate of closure (RROC), adapted from Tau theory, was the most significant predictor of the proportion of goals conceded. A validation process evaluated the new xG model for *CleanSheet* by comparing its difficulty predictions against user performance data across players of varying skill levels. CSxG effectively predicted shot difficulty at the extremes but showed less accuracy for mid-range scores (0.4 to 0.8). Additional variables influencing shot difficulty, such as build-up play and goalpost size, were identified for future model enhancements. This research contributes to the advancement of predictive modeling in sports performance analysis, highlighting the potential for improved goalkeeper training and strategy development using VR technology.

## 1. Introduction

Statistical measures of human performance have become integral to modern sports analysis [1]. In football (soccer), one of the most widely recognized metrics is the expected goals (xG) model [2]. Indeed, the concept of “expected goals” (xG) in football (soccer) has roots that trace back to early video game culture, where player and game performance metrics were popularized. While there is some debate over who first coined the term [3,4], xG gained widespread recognition through video games that focused on simulating realistic match conditions and probabilities [5]. Over time, xG has shifted from a niche analytic tool to a mainstream part of football analysis, though it remains somewhat misunderstood. While many analysts use xG to gauge the quality of scoring chances, there is ongoing debate about its broader predictive power, such as its potential to forecast match outcomes [3,4].

Simply put, xG is a measure of the quality of a shot and the likelihood of it resulting in a goal [2,6,7]. For instance, a shot with an xG value of 0.65 has a 65% chance of being converted into a goal. Numerous companies, including WyScout, Opta Analyst, and StatsBomb, have developed their own versions of xG using machine learning techniques such as logistic regression and XGBoost [8]. These xG scores are calculated the moment a shot is taken, factoring in variables such as shot location, proximity to defenders, and the body part used to strike the ball. For example, an Opta Analyst xG value of 0.85 would indicate that these factors combine to give an 85% probability of scoring a goal. Given its design, xG is primarily used to evaluate attacking players’ performance. Indeed, football pundits, and sometimes coaches, often judge players who frequently miss high-xG opportunities as underperforming while classifying players who consistently convert low-xG chances as exceptional finishers.

To accurately assess goalkeeping performance, it is essential to consider not only the outcomes (saves or goals conceded) but also the difficulty of each shot faced. Traditional performance metrics like the number of saves, GPS tracking of dives, or movement analysis lack vital information about ball trajectory and shot difficulty. In traditional football analytics, xG metrics help quantify shot difficulty, which aids in evaluating scoring opportunities and player decisions. That being said, xG ratings do not account for events after the attacking player has struck the ball and are therefore limited in their ability to assess goalkeeper performance. For instance, a goalkeeper saving a shot with an xG of 0.9 may appear to have made an impressive save, but if the attacker strikes the ball poorly—directly at the goalkeeper or off-target—the lack of a goal reflects poor attacking performance rather than exceptional goalkeeping. To fairly evaluate goalkeepers, a *post-shot* xG metric, which considers the ball’s trajectory, is necessary. While less common than standard xG, post-shot metrics like Stats Perform’s expected goals on target (xGOT) and StatsBomb’s post-shot xG (PSxG) have been developed. These metrics incorporate ball flight data, particularly shot placement, alongside the original xG to assess the likelihood of a goal after the shot is taken. Shots aimed at the corners of the goal, for example, tend to yield higher post-shot xG values than those directed towards the center [9].

*CleanSheet* allows users to play as a football (soccer) goalkeeper in a virtual reality (VR) environment. Users face sets of 10 shots from different set play scenarios. Avatars, animated using motion capture, represent the attacking and defending players. After a few passes, an attacking player shoots the ball towards the goal. As each of these shots has a different level of difficulty, the primary aim of this research is to develop a “CleanSheet xG” (CSxG) metric that can be used to quantify the probability that this shot would lead to a goal. If developed correctly, the CSxG metric should accurately classify shots that are more difficult to save, which would help identify the best-performing players (i.e., the goalkeepers who are able to save the difficult shots).

Currently, players can only track their performance in *CleanSheet* by looking at the total number of saves made rather than a metric that accounts for the difficulty of the shots they save (or concede). Players at the top of leaderboards may be the most skilled but could also be players who faced a higher number of shots with lower levels of difficulty (shots are randomly presented each time the player plays a session). A CSxG metric could overcome this issue by introducing leaderboards that incorporated this measure of the probability of “expected number of goals conceded” and use it to generate a more comprehensive goalkeeping performance score. For example, playing one 10-shot session where the mean CSxG is 0.58 would lead to an expected figure of 5.8 conceded goals for that session. On the flip side, a session with a mean CSxG of 0.86 would lead to 8.6 expected goals conceded. If one player conceded seven goals in the *0.58 CSxG session*, and another player conceded seven goals in the *0.86 CSxG session*, comparing the expected number of conceded goals to the actual number of goals conceded would identify the second player as being more skilled despite both players conceding the same number of goals. These data on expected vs. actual goals conceded can then be used to create future leaderboards that correctly identify the most skilled players [10].

## 2. Materials and Methods

### Data Wrangling

Being able to measure performance in any sport is very important for player development. It helps to identify areas of strength but also areas for improvement. The xG metric is no different and is extensively used in modern-day football. For example, expected goals (xG) is a metric used to assess the quality of scoring opportunities, providing insights into team and player performance by quantifying the likelihood of each shot resulting in a goal, and it is widely used to evaluate team effectiveness, predict outcomes, and identify players’ shooting efficiency. By comparing the actual results to the xG, analysts can gauge whether teams or players are over- or under-performing, making it a valuable tool for scouting, coaching, and predictive modeling.

Current xG models are often trained using thousands of historic shots [11], using the label of “goal” or “no goal” as the target. This is known as a binary classification task [12,13], where the model’s output is the probability of an instance belonging to one of two possible classes which, in this case, is the probability that a shot is a goal or not. This approach can be followed in a real-life scenario as no two shots will be identical across all input features. Some shots may be very similar, but the chances of an identical shot occurring, across the numerous variables used for xG models, is very low. As such, each shot can be assigned a binary label which is used as the target to train a classification model with no duplicate entries.

However, although suitable for classifying real match shots, this approach is not appropriate for CSxG. *CleanSheet*’s Set Play game mode has 936 pre-programmed shots, some of which have been played more than 50,000 times since user data have been captured. If every instance of a shot was used, with a success/failure label applied to train a classification model, this would lead to many duplicate entries across input features with differing labels. Using a regression model, where the target variable is the proportion, ranging from 0 to 1 (no goal (save) = 0; goal = 1) for instances where each shot played resulted in a goal or not, is more suitable. For example, a shot (hereafter routine) with a goal proportion of 0.8 indicates that a goal was conceded in 80% of the instances it was played by all players, and a save was made in 20% of all the instances played.

Once the goal proportion was calculated for each shot, it was necessary to try and understand what characteristics of that shot were making it difficult to save. To achieve this, JSON files containing the ball flight information, such as the start position, end position, angular velocity, and flight duration, were converted to a pandas dataframe. User data were collected from over 200,000 game play sessions and were stored as a CSV file. This was also converted to a pandas dataframe containing 3 columns—(i) the unique routine ID, (ii) a Boolean value referring to whether each shot resulted in a goal, and (iii) the number of times each shot had been played by users. Grouping this dataframe by routine ID led to a dataframe containing 936 entries, corresponding to the 936 playable shots available to save in Set Play. While grouping by routine ID, the mean of the Boolean entries for “goals conceded” was used to calculate the goal proportion for each routine. These final user data were joined with the ball flight dataframe using routine IDs, leading to a dataframe containing all ball flight information, as well as the goal proportion and number of times each routine was played.

It was decided that shots played a minimum of 100 times would be used for analysis. This was to ensure the validity of the goal proportion values. It was deemed that 100 instances were enough to ensure plays by multiple distinct users, as well as to overcome potential inconsistencies caused by factors external to the game such as glitches with the VR headset and controllers. Filtering for at least 100 plays reduced the dataset used for further analysis by 622 routines, and the resulting model was applied to the remaining 314 routines to produce an xG score for all routines within Set Play.

## 3. Results

### 3.1. Exploratory Data Analysis and Feature Engineering

Statistical techniques were used to check for outliers in ball flight data, such as shot entry location, flight duration, shot speed, and distance traveled. Outliers were identified using a standard method based on the range between the first and third quartiles (IQR). After reviewing these outliers, it was decided that no adjustments were needed. The outliers represented shots with deliberately extreme characteristics (very high or low speeds, for instance), which were intentionally included during development. Keeping these values ensured that the CSxG metric would accurately reflect the full range of shot difficulty, ranging from very easy to very challenging for a goalkeeper to save.

**Figure 1:**
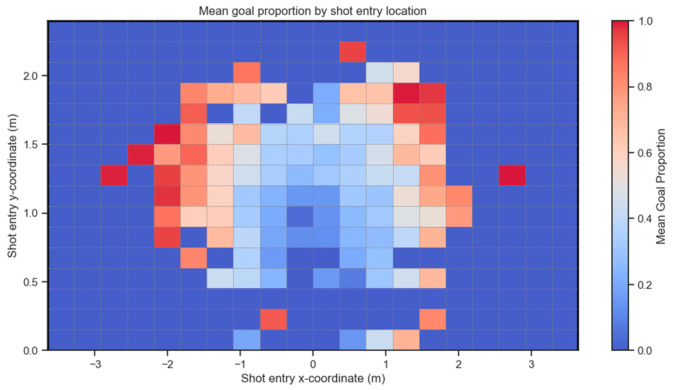
This heatmap shows the x and y coordinates of the goal mouth and the mean proportion of shots that ended up at the different locations in the goal. The goal mouth is 7.32 m wide and 2.44 m high (identical to the goal on a real soccer field). The spectrum represents the mean proportion of shots that resulted in a goal and ranges from red to dark blue (red = 1.0 and dark blue = 0.0 (no shot entered that zone)).

Existing post-shot xG metrics rely heavily on shot placement, i.e., the location of the ball within the goal face as it crosses the goal line [9]. Shots placed closer to the corners of the goal are deemed more difficult to save and thus have higher post-shot xG scores than shots closer to the center of the goal, which are classed as easier to save. Figure 1 shows the mean goal proportion for shots within Set Play grouped by shot placement. The limits of the axes represent the actual size of the goal mouth used in professional soccer, which is also the largest goal size available in the *CleanSheet* VR app. The goal mouth is split into 20 columns and 16 rows using the same measurements currently in use when shots are created for the game. The color bar on the right-hand side shows the colors corresponding to different goal proportion values, with a goal proportion of 1.0 meaning it always results in a goal. No shots recorded had a goal proportion of zero. Any areas of the goal mouth with the corresponding dark blue color, indicating a goal proportion of 0.0, represent areas of the goal where no shots enter. What is clear from Figure 1 is the positive relationship between the distance from the center of the goal and goal proportion, as discussed below.

The x and y coordinates representing the position where the shots crossed the goal line were used to calculate the Euclidean distance from the center of the goal for each routine. The center of the goal is defined as the point where x = 0 and y = 1 (where x = 0 represents the center of the goal mouth and y = 1 is 1 m above the center of the goal line). Previous research [12,14] found that shots with the lowest probability of resulting in a goal entered the goal face in the middle of the goalposts and close to the goalkeeper’s midriff. This study was based on professional goalkeepers in the English Premier League, and the height classified as “close to goalkeeper midriff” was 1.3 m above the ground. As a large proportion of CleanSheet’s users are under the age of 18, the midriff height was lowered to 1 m (y = 1). The resulting Euclidean distance was found to have a moderately strong positive Pearson’s correlation coefficient (r) of 0.698 with goal proportion.

In the same study [14], Gelade discussed the impact that the position on the pitch that the shot is taken from had on the probability of a save being made. Shots taken closer to goal are likely to reach the goal line quicker, giving the goalkeeper less time to act, and giving a lower probability of the shot being saved. Two shots entering identical areas of the goal face will vary in difficulty to save if this “time to act” is different for the two shots. To account for this, a spatiotemporal variable, combining the distance from the center of the goal and time to act, was explored for each shot.

Many actions in sports, such as catching a ball in a rugby line-out, hitting a baseball pitch with a bat, or intercepting a soccer ball before it crosses the goal line, can be explained by general Tau theory [15,16]. General Tau theory provides a parsimonious yet elegant solution for intercepting moving objects. In simplistic terms, the variable Tau (τ), or time to closure, is defined as follows:

(1) $$\tau =\frac{x\;(Size\;of\;gap\;to\;close)}{\dot{x}\;(Current\;rate\;of\;gap\;closure)}$$

In *CleanSheet*, the player must close the gap between their hand(s) at the time the shot is struck and the location the ball will cross the goal line in the time that it takes the ball to reach that point. Taking this into account, the above equation can then be framed as follows:

(2) $$\tau \;(Time\;to\;closure/flight\;duration)=\frac{Distance\;from\;centre}{Required\;rate\;of\;closure\;(RROC)}$$

In turn, it can be rearranged as follows:

(3) $$Required\;rate\;of\;closure\;(RROC)=\frac{Distance\;from\;centre\;goal}{\tau \;(flight\;duration)}$$

**Figure 2:**
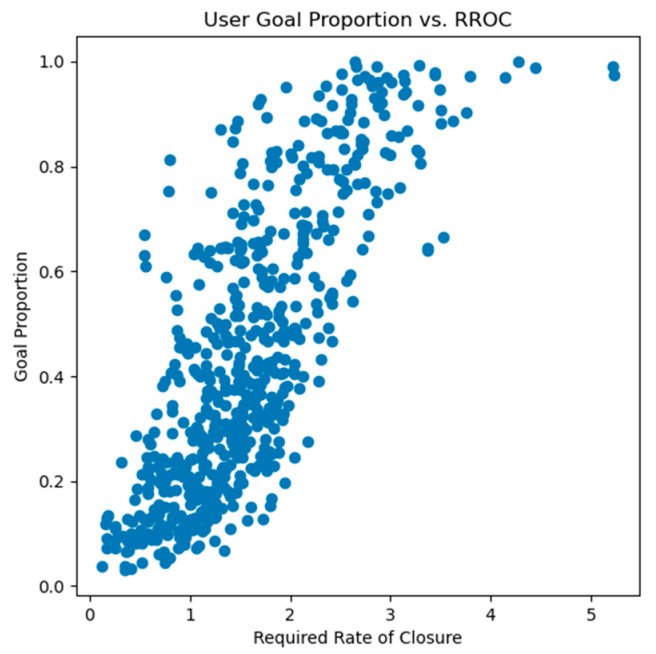
This figure shows a strong positive correlation between the required rate of closure (adapted from General Tau theory) of the shots faced and the proportion of goals conceded during CleanSheet game play (r = 0.778).

The flight duration (included in the metadata) and Euclidean distance from the center were then used to calculate an RROC for each shot. When looking at the relationship between the RROC and proportion of goals conceded, a strong positive correlation of r = 0.778 was found (see Figure 2). The RROC was therefore selected as an input feature for the CSxG model instead of using the distance from the center in isolation.

Other research has also shown how spin-induced deviations in a ball’s trajectory can negatively impact a player’s ability to anticipate where the ball will go [14]. Spin-induced deviations in ball flight can therefore increase the likelihood of a shot resulting in a goal [17,18]. Like any interceptive action, stopping a shot requires the goalkeeper to anticipate the ball’s arrival position [17]. Based on the ball’s apparent heading direction, shots with spin often cause goalkeepers to initially move in the wrong direction [18]. As a result, the spin-induced deviation of a ball’s flight path was also included as a key input feature in this study. To calculate this, initial velocity vectors and coordinates for the shot’s launch and end positions were used. Newtonian equations of motion were applied to estimate the x (lateral) coordinate when crossing the goal line in the absence of spin. The difference between this value and the actual x coordinate at the point of entry was used to measure shot curvature. A weak positive correlation between curvature and goal proportion was observed.

The final input feature, commonly used in traditional pre-shot xG models, is the angle between the shooter and the center of the goal. Shots taken from tighter angles have less of the goal to aim at and are typically a key factor in xG models [2,12,14]. In our case, a weak negative correlation was observed between the shot angle and goal proportion, indicating that as the angle becomes tighter, the likelihood of scoring decreases. An Ordinary Least Squares (OLS) regression analysis confirmed that the three variables discussed—RROC, spin, and shot angle (see Table 1)—had a statistically significant impact on goal proportion, with the *p*-values for all input coefficients being below 0.05.

### 3.2. Model Selection

A variety of models, such as logistic regression, random forest, XGBoost, neural networks, and AdaBoost, have been employed to generate existing expected goals (xG) metrics [2,19,20]. While these models can be applied to both regression and classification tasks, their suitability for constructing the CSxG model was limited for several reasons. First, classification-based approaches were not appropriate, as they could lead to identical CSxG scores for shots with similar input features, whereas distinct scores for each shot were required. Second, the limited dataset and small number of features posed a significant risk of overfitting with highly complex models—a risk confirmed in the case of XGBoost. Third, we prioritized creating a model that was transparent and interpretable to a non-technical audience, avoiding the “black box” nature of certain machine learning methods [21,22]. Nevertheless, some of these more complex techniques were used to validate feature importance, as detailed in the Section 3 below. Ultimately, a regression model was selected to balance simplicity, interpretability, and performance.

An initial “level of difficulty” metric was created, incorporating a linear combination of shot velocity, curvature, and flight duration. While the weights were derived from domain knowledge, the metric ultimately performed poorly when compared to user data. The level of difficulty metric exhibited a Pearson correlation coefficient of 0.246 with the proportion of goals conceded in the user data. As previously discussed, shot placement is a crucial factor in determining a shot’s difficulty to save, and this was not accounted for in the initial model. Another issue was the unbounded nature of the output. To address this, the results were “winsorized”, with values greater than 1 capped at 1 [23]. However, this adjustment led to a significant discrepancy between the predicted difficulty level and the observed user data. For example, shots with a difficulty rating of 1 (indicating maximum difficulty) showed user goal-conceded proportions ranging from 0.06 to 0.99.

While the OLS regression method showed a linear relationship between the chosen input variables and goal proportion, a linear model would face the same issue of unbounded results as the level of difficulty discussed above. Logistic regression, commonly used for xG models, has outputs bounded between 0 and 1 as desired due to a sigmoidal activation function [24]. While logistic regression is unsuited to our data, the sigmoid function can be adapted for use in CSxG.

The logistic (sigmoidal) function of the form is as follows:

(4) $$f\left(x\right)=\frac{1}{\frac{1}{u}+ab^{x}}$$

where *u* is the upper bound of the target, x is the input, and *a* and *b* are optimized constants found in many studies featuring gap closure and the Tau theory [12,13]. The following was adapted to obtain the function used to find CSxG:

(5) $$CSxG=\frac{1}{1+ab^{X}}$$

where *X* = *c* x RROC + *d* x curvature + *e* x shot angle, with *a*, *b*, *c*, *d*, and *e* being constants to be optimized, with an upper bound of 1 leading to $\frac{1}{u}$ from the initial function to equal 1. “Curve_fit” from the scipy package, using the iterative Levenberg–Marquardt algorithm, was used to optimize the constants, minimizing the sum of squared residuals for our nonlinear model [25]. Five-fold stratified cross validation was used to assess the generalizability of the function. The function was then fitted to the 622 shots used for analysis to calculate the values of the constants, with this final model applied to the remaining shots to find CSxG scores for all routines.

### 3.3. Evaluating the Model Mathematically

The metrics selected to evaluate the performance of the CSxG model were the coefficient of determination (R^2^) and root mean square error (RMSE). R^2^ ranges from 0 to 1 indicate how much of the variability in the dependent variable is explained by the regression model [26]. A value of 1 represents 100% explained variability, while a value of 0 indicates no explained variability. RMSE is the square root of the mean value of residuals, where residuals represent the difference between observed values and their corresponding predictions [27].

**Figure 3:**
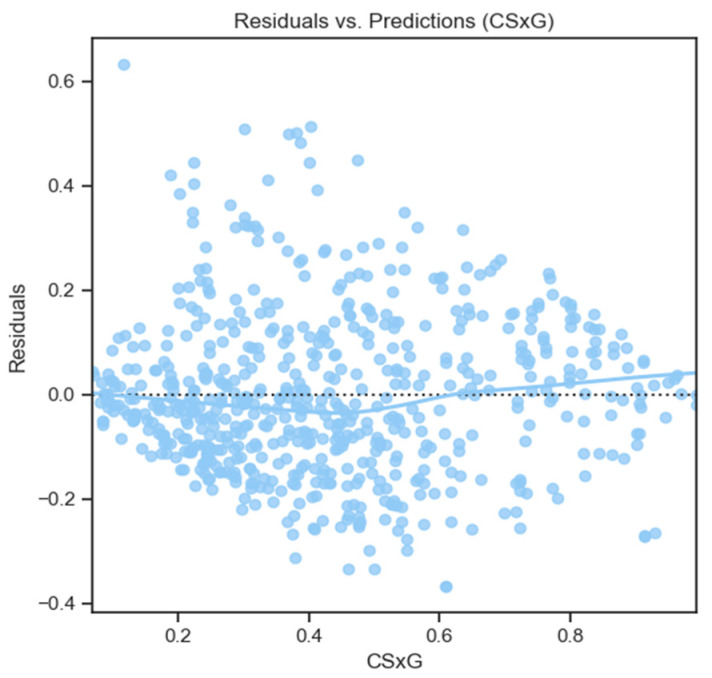
This figure shows the relationship between the residuals and the CSxG ratings for all of the different *CleanSheet* shots.

The custom CSxG function incorporated the input features discussed previously, with the user goal proportion for each shot serving as the target. The model displayed no evidence of overfitting. During cross-validation, the mean training and test R^2^ values were 0.660 and 0.651, respectively, while the mean training and test RMSE values were 0.155 and 0.157. The standard deviations for training and test R^2^ were 0.002 and 0.01, respectively, and for RMSE, they were 0.0007 and 0.003, indicating consistent performance across all folds (see Figure 3).

The same CSxG function was fitted using scaled input features via scikit-learn’s “StandardScaler”, which standardized all features to have a mean of 0 and a standard deviation of 1 [28]. This led to nearly identical performance, with the mean training R^2^ value remaining unchanged and the mean test R^2^ value increasing by less than 0.01 during cross-validation. However, this scaling made the relative importance of features more transparent compared to the model with unscaled inputs. Among the features, RROC (Required Rate of Closure) was the most heavily weighted, with a coefficient approximately five times larger in magnitude than those for curvature and shot angle, which had very similar weightings.

**Figure 4:**
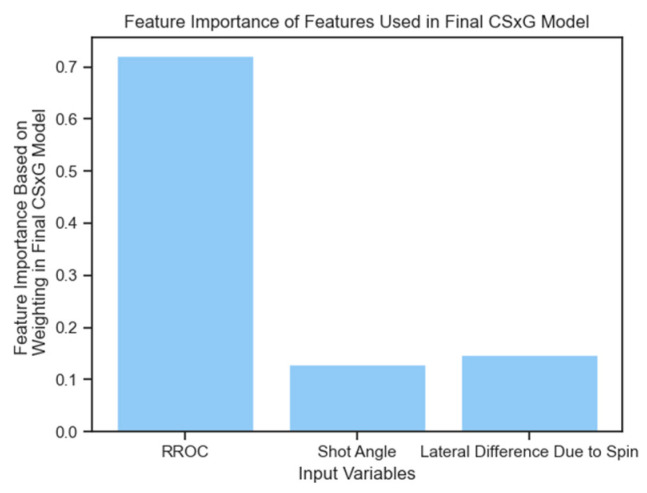
A bar graph showing feature importance based on weighting in the final CSxG model. It can be noted that the RROC variable was the most salient.

**Figure 5:**
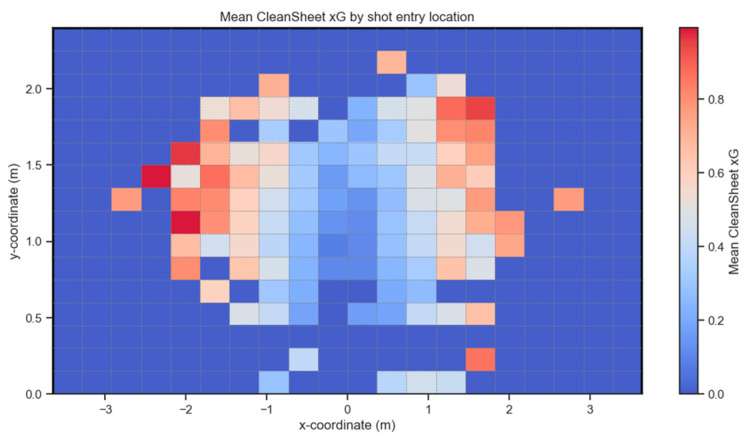
A heatmap showing the results of the validation of the CSxG metric and the shot entry location. The pattern shown was very similar to that observed from real gameplay in Figure 1 for the mean proportion of goals conceded for the different goal locations.

To further validate these feature importances, alternative models such as XGBoost, a gradient boosting regressor, and a decision tree were employed. In all cases, RROC emerged as the most significant feature, with feature importance scores of 0.73 and 0.79 in XGBoost and the gradient boosting regressor, respectively (see Figure 4). As discussed in the feature engineering section (Section 3.1), RROC is influenced by both shot placement within the net and the time to act (i.e., flight duration of the shot). Figure 5 presents a heatmap of the mean CSxG for shots grouped by shot placement. Given that shot placement plays a central role in RROC, which, in turn, is a key factor in CSxG, it is unsurprising that this heatmap shows similar patterns to those presented in Figure 1 and recorded from thousands of sessions of gameplay.

### 3.4. Evaluating the Model Using Human Performance Data

**Figure 6:**
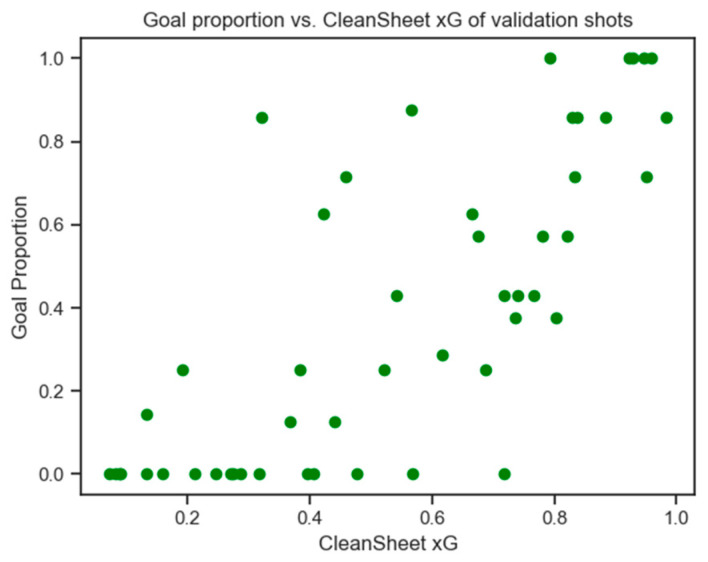
A scatter graph showing the relationship between the new CSxG metric and the proportion of goals conceded by the group of 6 players.

Validation of the CSxG metric was conducted using a set of 50 shots selected based on their CSxG ratings. Six participants, comprising novice (n = 3) and experienced (n = 2) CleanSheet users, along with one semi-professional goalkeeper (n = 1), attempted to save the shots. A strong correlation of 0.771 was observed between the CSxG scores and the goal proportions for these validation shots. Shots predicted as very easy (CSxG near 0) or very difficult (CSxG near 1) to save aligned well with the actual proportions of goals conceded. However, CSxG ratings between 0.4 and 0.8 showed more variability. Figure 6 illustrates the relationship between CSxG and proportions of goals conceded from six participants during the validation process.

## 4. Discussion

A conventional xG metric, while valuable for assessing shot quality, falls short in evaluating goalkeeping performance because it does not consider crucial ball flight parameters that influence a goalkeeper’s ability to make a save. This research aimed to develop a novel CSxG rating system for the VR goalkeeping simulator, *CleanSheet*, to provide a more accurate measure of goalkeeper performance based on shot difficulty. A novel CSxG metric was constructed using current xG concepts, known factors that influence goalkeeper performance and predictive modeling. The CSxG model was then regressed against the data on conceded goals harvested from CleanSheet gameplay (>100,000 users). This regression model allowed us to assign CSxG scores to existing shots which, in turn, could be used to assign CSxG scores to new, future shots. Performance metrics such as the coefficient of determination (R2) and the root mean squared error (RMSE), as well as examining the residuals and conducting a validation process, showed that the model had very good predictive validity for shots with difficulty levels at the extreme ends of the scale (r = 0.771, explaining over 77% of the variance). In other words, the CSxG metric could identify shots saved by users very often (very low CSxG values) along with shots that were very rarely saved by users (very high CSxG value). As expected with such models, CSxG values in the middle range of 0.4 to 0.8 had less predictable outcomes.

Although the model had good predictive validity, over 20% of the variance was still not explained by the model. A feature that could explain some of this could be the size of the goal selected during gameplay (note that different goal widths and heights can be selected by users in *CleanSheet*). A larger goal size could result in a larger distance the player has to cover when making a save (in other words, the area the striker can aim at is much larger) compared to a smaller goal size. These extra options could lead to players finding a shot difficult to save with the largest goal size despite finding the same shot being straightforward to save with the smallest goal size [14].

Another variable that could be included in a future model to improve the predictive validity is the shot angle. Indeed, many current xG models include the shot angle as an important variable. For instance, shots taken from the center of the pitch increase the options for a striker (i.e., they can go left or right), giving goalkeepers more areas of the goal to consider covering effectively [2,12,14].

Another factor that will account for some of the unexplained variance in goals saved by the CSxG is the inherent variability in player abilities. More experienced or talented goalkeepers are likely to optimize their positioning at the time of a shot [29,30] and have greater agility, faster reaction times, and potentially taller physiques with larger arm spans, allowing them to cover more of the goal without needing to cover as much distance as other goalkeepers [31,32,33]. While this positional and physical data could be captured by the VR system and used to improve predictive accuracy, incorporating player ability into the model could distort its effectiveness in identifying skill levels. For instance, a highly skilled goalkeeper could position themselves optimally, reducing the CSxG score from 0.8 (based on shot characteristics) to 0.3, making it appear as though they saved an easy shot, when in fact the shot would have been difficult for most players. This would result in an underestimation of their true ability. Consequently, positional and physical attributes are unlikely to be integrated into future CSxG models.

Although CSxG was generated to quantify the level of difficulty of shots in a VR app, it has real-world implications for football statistics and performance analysis more generally. A key limitation in traditional xG and PSxG models is the absence of detailed post-shot ball flight data and the time a goalkeeper has to respond. By incorporating a novel variable, the Required Rate of Closure (RROC), which blends the ball arrival position as a function of the distance from the goal center and the time it will take for the ball to arrive there, the CSxG provides a more nuanced representation of shot difficulty. These enhancements could significantly refine real football xG models by better accounting for the constraints (namely the ball’s arrival position and shot duration) that ultimately dictate the time within which a goalkeeper must perform their action.

By establishing an objective measure of shot difficulty, our CSxG model can serve as a foundation for assessing goalkeeping ability separately. This could lead to the development of an “expected saves” (xS) metric, which would more accurately evaluate goalkeepers’ performance. Improving existing xG models and integrating a new performance metric such as xS into real-world football data pipelines would allow analysts and coaches to evaluate not just scoring probabilities but also goalkeeping effectiveness, offering a more holistic understanding of match dynamics and player performance.

## 5. Conclusions

Video games have played a pivotal role in popularizing the concept of expected goals (xG), now a staple in football analysis, which helps pundits assess shot quality and scoring probability. In this study, we developed a novel expected goals (xG) metric, CSxG, for use in a VR-based goalkeeping simulator to quantify player (goalkeeper) performance. Using sensor data from the VR environment, we found strong agreement between our CSxG model and actual goalkeeping performance, particularly for shots predicted as being very easy or very difficult to save. This model provides valuable insights into player ability, offering a potential tool for identifying talented goalkeepers in both virtual and realistic performance-based environments. In conclusion, the CSxG metric developed in this study quantifies the difficulty of a shot without confounding the variable with the goalkeeper’s skill or positioning.

## Figures and Tables

**Table 1:** This table provides details of the final features used in the CSxG model.

Model Feature	Description
**Required Rate of Closure (RROC)**	Derived from the Tau theory [15], this measures the time needed to close the gap between the ball and the goal. It is calculated as the Euclidean distance from the center of the goal (0,1) to the point where the ball crosses the goal line (x,y) divided by the time that passes until the ball reaches the goal.
**Shot Angle**	This is the lateral angle (0–90° in both directions) between the center of the goal and the shot’s origin on the pitch. A shot from the penalty spot has an angle of 0°, while a shot from the corner flag has an angle of 90°.
**Lateral Difference Due to Spin**	This is the lateral displacement, in meters, between where the ball crosses the goal line and where it would have crossed without spin. It is calculated using initial velocity vectors and Newtonian equations of motion to predict the spin-free trajectory of the ball.

## Data Availability

The data supporting this study are not publicly available due to their commercially sensitive nature. However, the data may be made available upon reasonable request to the corresponding author along with the execution of a Material Transfer Agreement to ensure proper use and confidentiality.

## References

[1] Severini T.A. (2020). Analytic Methods in Sports: Understanding Mathematics and Statistics to Understand Data from Baseball, Football, Basketball and Other Sports.

[2] Mead J., O’Hare A., McMenemy P. (2023). Expected goals in football: Improving model performance and demonstrating value. PLoS ONE.

[3] Barnett V., Hilditch S. (1993). The effect of an artificial pitch surface on home team performance in football (soccer). J. R. Stat. Soc. Ser. A Stat. Soc..

[4] Ensum J., Pollard R., Taylor S. (2004). Applications of logistic regression to shots at goal in association football: Calculation of shot probabilities, quantification of factors and player/team. J. Sports Sci..

[5] Parkin S. Fifa: The video game that changed football. The Guardian.

[6] Spearman W. Beyond expected goals. Proceedings of the 12th MIT Sloan Sports Analytics Conference.

[7] Hewitt J., Karakus O. (2023). A machine learning approach for player and position adjusted expected goals in football (soccer). Frankl. Open.

[8] Whitmore J. What Is Expected Goals (xG)?. https://theanalyst.com/2023/08/what-is-expected-goals-xg/.

[9] Whitmore J. What Are Expected Goals on Target (xGOT)?. https://theanalyst.com/eu/2021/06/what-are-expected-goals-on-target-xgot/.

[10] Velez J.A., Ewoldsen D.R., Hanus M.D., Song H., Villarreal J.A. (2018). Social comparisons and need fulfilment: Interpreting video game enjoyment in the context of leaderboards. Commun. Res. Rep..

[11] Cavus M., Biecek P. Explainable expected goal models for performance analysis in football analytics. Proceedings of the 2022 IEEE 9th International Conference on Data Science and Advanced Analytics (DSAA).

[12] Pollard R., Ensum J., Taylor S. (2004). Estimating the probability of a shot resulting in a goal: The effects of distance, angle and space. Int. J. Soccer Sci..

[13] Canbek G., Sagiroglu S., Temizel T.T., Baykal N. Binary classification performance measures/metrics: A comprehensive visualized roadmap to gain new insights. Proceedings of the 2017 International Conference on Computer Science and Engineering (UBMK).

[14] Gelade G. (2014). Evaluating the ability of goalkeepers in English Premier League football. J. Quant. Anal. Sports.

[15] Lee D.N., Georgopoulos A.P., Clark M.J., Craig C.M., Port N. (2001). Guiding contact by coupling the taus of gaps. Exp. Brain Res..

[16] Watson G., Brault S., Kulpa R., Bideau B., Butterfield B., Craig C.M. (2010). Judging the ‘passability’ of dynamic gaps in a virtual rugby environment. Hum. Mov. Sci..

[17] Craig C.M., Bastin J., Montagne G. (2011). How information guides movement: Intercepting curved free kicks in soccer. Hum. Mov. Sci..

[18] Dessing J.C., Craig C.M. (2010). Bending it like Beckham: How to visually fool the goalkeeper. PLoS ONE.

[19] Garau G. Big Data in Soccer: Creating an xG Model. https://blog.damavis.com/en/big-data-in-soccer-creating-an-xg-model/.

[20] Anzer G., Bauer P. (2021). A goal scoring probability model for shots based on synchronized positional and event data in football (soccer). Front. Sports Act. Living.

[21] Hawkins D.M. (2004). The problem of overfitting. J. Chem. Inf. Comput. Sci..

[22] Rudin C. (2019). Stop explaining black box machine learning models for high stakes decisions and use interpretable models instead. Nat. Mach. Intell..

[23] Blaine B.E. (2018). Winsorizing. The SAGE Encyclopedia of Educational Research, Measurement, and Evaluation.

[24] Zou X., Hu Y., Tian Z., Shen K. Logistic Regression Model Optimization and Case Analysis. Proceedings of the IEEE 7th International Conference on Computer Science and Network Technology (ICCSNT).

[25] Ranganathan A. (2004). The Levenberg-Marquardt Algorithm. Tutor. LM Algorithm.

[26] Nagelkerke N.J.D. (1991). A note on a general definition of the coefficient of determination. Biometrika.

[27] Hodson T.A. (2022). Root-mean-square error (RMSE) or mean absolute error (MAE): When to use them or not. Geosci. Model Dev..

[28] Pedregosa F., Varoquaux G., Gramfort A., Michel V., Thirion B., Grisel O., Blondel M., Prettenhofer P., Weiss R., Dubourg V. (2011). Scikit-learn: Machine learning in Python. J. Mach. Learn. Res..

[29] Goodman M. A New Way to Measure Keepers’ Shot Stopping: Post-Shot Expected Goals. https://statsbomb.com/articles/soccer/a-new-way-to-measure-keepers-shot-stopping-post-shot-expected-goals/.

[30] Lamas L., Drezner R., Otranto G., Barrera J. (2018). Analytic method for evaluating players’ decisions in team sports: Applications to the soccer goalkeeper. PLoS ONE.

[31] Hughes M., Caudrelier T., James N., Redwood-Brown A., Donnelly I., Kirkbride A., Duschesne C. (2012). Moneyball and soccer—An analysis of the key performance indicators of elite male soccer players by position. J. Hum. Sport Exerc..

[32] Perez-Arroniz M., Calleja-Gonzalez J., Zabala-Lili J., Zubillaga A. (2021). The soccer goalkeeper profile: Bibliographic review. Physician Sportsmed..

[33] Otte F.W., Millar S.-K., Klatt S. (2019). How does the modern football goalkeeper train?—An exploration of expert goalkeeper coaches’ skill training approaches. J. Sports Sci..

